# Intermittent versus continuous enteral nutrition in critically ill patients: an updated systematic review and meta-analysis of randomized controlled trials

**DOI:** 10.3389/fnut.2026.1786580

**Published:** 2026-04-21

**Authors:** Shu Zhang, Shuxia Yu, Kai Zhang, Honghang Lin

**Affiliations:** 1 Department of Emergency Medicine, The First People’s Hospital of Taizhou, Taizhou, China; 2 Department of Cardiothoracic Surgery, The First People’s Hospital of Taizhou, Taizhou, China; 3 Department of Critical Care Medicine, Second Affiliated Hospital, Zhejiang University School of Medicine, Hangzhou, China

**Keywords:** continuous enteral nutrition, critically ill, intermittent enteral nutrition, meta-analysis, nutrition support

## Abstract

**Background:**

Both intermittent enteral nutrition (IEN) and continuous enteral nutrition (CEN) are used to provide nutritional support to critically ill patients. However, their comparative effects on gastrointestinal tolerance and clinical outcomes remain uncertain.

**Objectives:**

We conducted an updated systematic review and meta-analysis of randomized controlled trials (RCTs) to compare the efficacy and safety of IEN versus CEN in critically ill patients.

**Methods:**

We performed a comprehensive literature search of PubMed, Embase, Scopus, and the Cochrane Library from inception through December 10, 2025, to identify RCTs comparing IEN and CEN in critically ill adults. The primary outcome was all-cause mortality in the intensive care unit (ICU). Secondary outcomes included gastrointestinal complications, length of ICU stay, and achievement of nutritional goals. Pooled risk ratios (RRs) and mean differences (MDs) with 95% confidence intervals (CIs) were calculated using random-effects or fixed-effects models as appropriate. Subgroup analyses were performed according to mechanical ventilation status.

**Results:**

Twenty-two RCTs comprising 1,662 critically ill patients (IEN, *n* = 816; CEN, *n* = 846) were included. Compared with CEN, IEN was associated with a significantly higher incidence of diarrhea (RR 1.56, 95% CI 1.23 to 1.98, *I*^2^ = 19%) and abdominal distension (RR 1.68, 95% CI 1.10 to 2.57, *I*^2^ = 18%), as well as prolonged ICU length of stay (MD 0.91, 95% CI 0.41 to 1.41, *I*^2^ = 0%). Conversely, IEN was associated with a lower incidence of constipation (RR 0.74, 95% CI 0.57 to 0.97, *I*^2^ = 0%). These effects were more pronounced in mechanically ventilated patients, whereas no statistically significant differences were observed in non-ventilated patients. No significant differences were identified between the two strategies regarding ICU mortality, vomiting, gastric retention, aspiration pneumonia, or achievement of nutritional goals.

**Conclusion:**

This updated meta-analysis demonstrates that IEN is associated with increased rates of diarrhea and abdominal distension and prolonged ICU length of stay compared with CEN, particularly among mechanically ventilated patients. Although IEN reduces the incidence of constipation, CEN may be the preferable feeding strategy for most critically ill patients. Individualized approaches considering patient-specific factors and clinical context are warranted. Further high-quality trials are needed to identify patient subgroups who might benefit from IEN.

**Systematic review registration:**

https://osf.io/krs8v.

## Introduction

Malnutrition and catabolic stress are prevalent among critically ill patients and are associated with increased infection rates, prolonged mechanical ventilation, and elevated mortality (1, 2). Early enteral nutrition (EN) is recommended as the preferred route of nutritional support when the gastrointestinal tract is functional, as it maintains gut integrity, modulates immune responses, and reduces infectious complications compared with parenteral nutrition (3, 4). However, the optimal method for delivering EN in the intensive care unit (ICU) remains a subject of ongoing debate (5).

Two primary strategies are widely employed in clinical practice: continuous and intermittent enteral feeding (6). Continuous enteral nutrition (CEN), defined as prolonged infusion typically administered over 18 to 24 h per day using feeding pumps, has traditionally been the preferred approach in ICU settings (7). This method theoretically provides steady nutrient delivery, maintains stable metabolic conditions, and minimizes fluctuations in blood glucose levels. In contrast, intermittent enteral nutrition (IEN) involves the administration of defined volumes of feeding formula over shorter periods several times daily, more closely mimicking the physiological pattern of normal oral intake (6). Emerging evidence suggests that IEN may offer certain advantages, including shorter time to achieve nutritional targets and potentially favorable metabolic effects in specific patient populations (8).

Current clinical practice guidelines present divergent recommendations regarding the optimal feeding strategy. The American Society for Parenteral and Enteral Nutrition (ASPEN) and the European Society for Clinical Nutrition and Metabolism (ESPEN) guidelines have traditionally favored continuous feeding for critically ill patients (9, 10). However, emerging data have prompted reconsideration of this approach. The Japanese Critical Care Nutrition Guideline 2024 acknowledged ongoing uncertainty, noting insufficient evidence to make strong recommendations favoring either strategy (4). Furthermore, a recent expert perspective challenged conventional practices by suggesting that daytime-intermittent nutrition support can be safely initiated and may offer practical advantages in ICU settings (11). This evolving landscape underscores the need for a comprehensive synthesis of available evidence.

Previous meta-analyses have yielded conflicting findings, resulting in a lack of consensus. Some studies suggest that continuous feeding may be associated with a lower incidence of diarrhea and abdominal distension (12, 13), whereas others indicate that intermittent feeding might reduce the risk of constipation (14). A recent meta-analysis demonstrated no clinically relevant differences in important clinical outcomes, such as mortality and ICU length of stay, between the two strategies (15). Given the accumulation of new evidence and the persistent clinical equipoise regarding the optimal EN delivery strategy, an updated and expanded meta-analysis is warranted to provide contemporary guidance for clinical practice. The present study aimed to systematically review and synthesize all available evidence comparing IEN and CEN strategies in critically ill adult patients.

## Methods

This updated systematic review and meta-analysis was based on a previously published review (13) and was conducted in accordance with the Preferred Reporting Items for Systematic Reviews and Meta-Analyses (PRISMA) 2020 guidelines (Supplementary Table S1) (16). The study protocol was prospectively registered with the Open Science Framework.^1^

### Search strategy and study selection

Relevant randomized controlled trials (RCTs) were identified through a comprehensive search of PubMed, Embase, Scopus, and Cochrane Library from database inception through December 10, 2025. The following keywords and their combinations were used: “enteral nutrition,” “continuous feeding,” “intermittent feeding,” “bolus feeding,” “critically ill,” and “randomized controlled trial”. The complete search strategies are provided in Supplementary Table S2.

Studies were included if they met the following criteria: (1) study design: RCTs; (2) population: adult critically ill patients (aged ≥ 18 years) requiring enteral nutrition; (3) intervention: IEN, including bolus or cyclic feeding; (4) comparator: CEN; (5) outcomes: at least one clinical outcome of interest, including gastrointestinal complications (diarrhea, abdominal distension, vomiting, constipation, gastric retention, or aspiration pneumonia), ICU mortality, ICU length of stay, or achievement of nutritional goals.

Studies were excluded if they: (1) exclusively enrolled pediatric patients; (2) were non-randomized studies, case reports, or review articles; (3) did not clearly define the enteral nutrition delivery method; (4) provided insufficient data for quantitative analysis; or (5) enrolled patients with specific gastrointestinal conditions precluding standard enteral nutrition protocols.

### Data extraction

Two independent reviewers performed the initial screening of titles and abstracts, subsequently proceeding to a full-text assessment of studies identified as potentially eligible. Any discrepancies between the reviewers were adjudicated either through mutual discussion or by soliciting the opinion of a third reviewer. Essential data were systematically extracted from each included study using a standardized form, capturing the following details: first author’s name, publication year, country of origin, sample size, participant characteristics (including age, sex, and disease severity), specific intervention parameters (such as feeding protocol, infusion rate, and duration), and relevant outcome measures. When studies reported continuous outcomes as medians and interquartile ranges (IQRs), we applied the methodology described by Wan et al. (17) to convert these data into means and standard deviations (SDs).

### Quality assessment

Two reviewers independently appraised the methodological quality of the included randomized controlled trials (RCTs) using the Cochrane Risk of Bias Tool, Version 2 (RoB 2.0) (18). This tool assesses the risk of bias across five key domains: the randomization process, deviations from intended interventions, missing outcome data, measurement of outcomes, and selection of the reported results. Each domain was categorized as having a “low risk,” “some concerns,” or “high risk” of bias. The overall risk of bias for each individual study was then derived from the collective ratings of these domains.

### Statistical synthesis and analysis

The meta-analyses were conducted utilizing Review Manager (RevMan) version 5.4 (The Cochrane Collaboration) and R statistical software version 4.0, incorporating the “meta” and “robvis” packages. For dichotomous outcomes, risk ratios (RRs) along with their 95% confidence intervals (CIs) were computed employing the Mantel–Haenszel method. Regarding continuous outcomes, mean differences (MDs) and corresponding 95% CIs were derived using the inverse variance method.

Statistical heterogeneity among the included studies was assessed with the I^2^ statistic (19). The magnitude of heterogeneity was interpreted based on established thresholds: an *I*^2^ value of 25% was considered low, 50% denoted moderate heterogeneity, and 75% indicated a high degree of heterogeneity. A random-effects model was used when substantial heterogeneity was present (*I*^2^ > 50%); otherwise, a fixed-effects model was applied. Publication bias was assessed through visual inspection of funnel plots for asymmetry and Egger’s linear regression test (20).

Sensitivity analyses were conducted by sequentially excluding individual studies (leave-one-out analysis) to assess the robustness of pooled results. Subgroup analyses were planned *a priori* according to mechanical ventilation status: patients were stratified into a mechanical ventilation subgroup and a non-mechanical ventilation subgroup.

## Results

### Study characteristics

**Figure 1:**
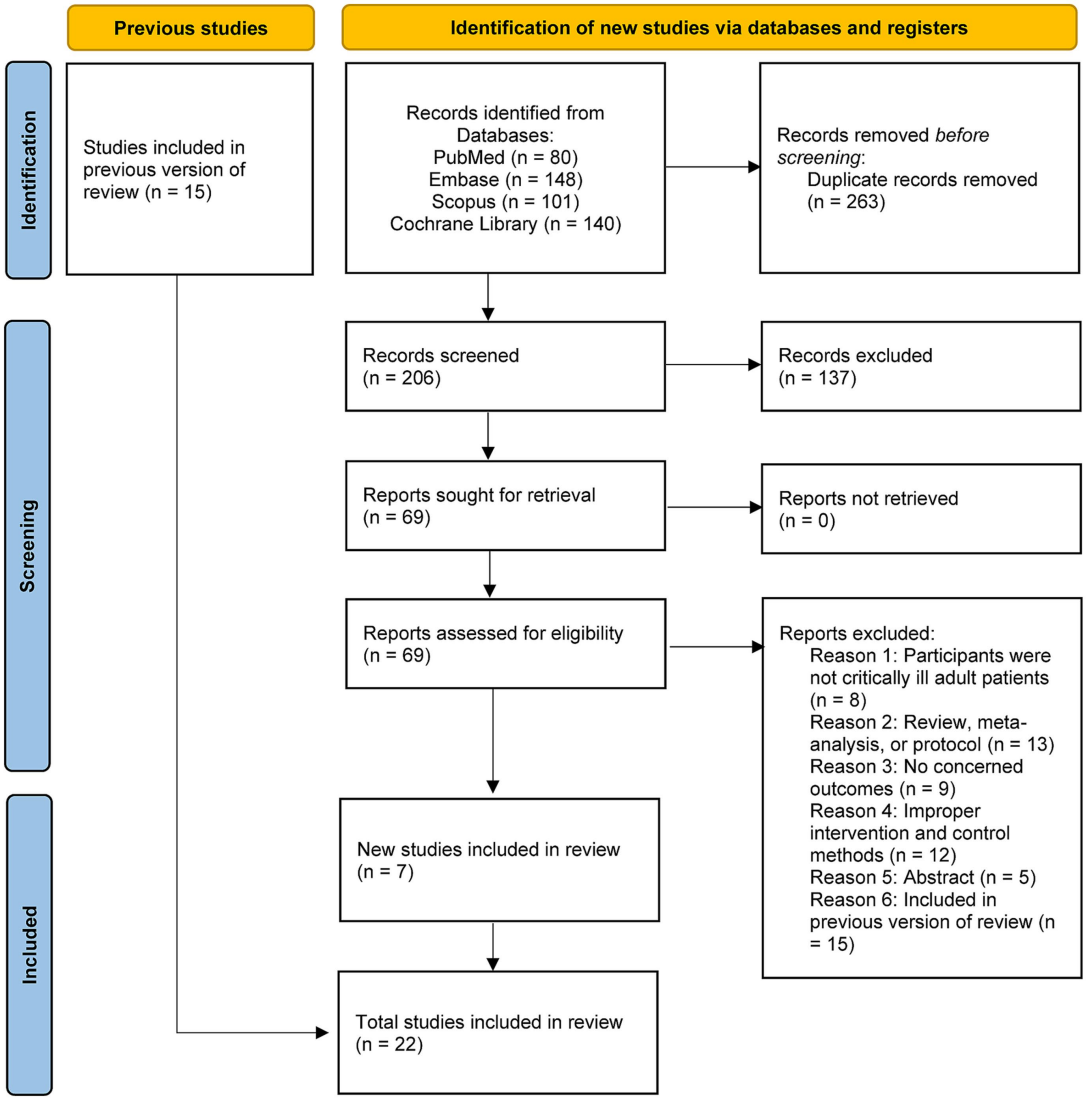
PRISMA 2020 flow diagram for this updated systematic review and meta-analysis.

The updated literature search identified 469 records. After removing duplicates, 206 records underwent title and abstract screening. Following full-text review of 69 potentially eligible articles, 22 RCTs (21–42) met the inclusion criteria and were included in this updated meta-analysis, representing an addition of 7 new RCTs (36–42) since our previously published review. The study selection process is illustrated in the PRISMA flow diagram (Figure 1).

The included studies were published between 1986 and 2025 and enrolled a total of 1,662 critically ill patients (816 receiving IEN and 846 receiving CEN). Patient populations varied across studies and included general ICU patients (*n* = 15 studies), surgical ICU patients (*n* = 3 studies), neurological ICU patients (*n* = 2 studies), and patients with sepsis (*n* = 2 studies). Across 13 studies, disease severity was quantified using the Acute Physiology and Chronic Health Evaluation (APACHE) II score, with mean values ranging from 12 to 29 points, reflecting intermediate to high illness severity.

Regarding feeding protocols, IEN protocols demonstrated considerable heterogeneity, with administration frequencies ranging from every 3 h to every 18 h, although 4-hourly delivery predominated. Notably, some IEN regimens involved prolonged infusion durations (up to 18 h per day), which may overlap conceptually with continuous feeding strategies and introduce clinical heterogeneity. CEN was administered continuously over 24 h in 20 studies, whereas 2 studies (33, 35) employed an 18-h daily infusion schedule. The duration of nutritional support ranged from 3 to 14 days, with most included trials evaluating outcomes over a 7-day observation period. Comprehensive study characteristics are presented in Table 1.

**Table 1:** Characteristics of included studies.

Study and year	Number of participants	Population	Characteristics (IEN/CEN)	Intervention and study period
Saiz-Vinuesa et al. (38)	IEN: 22; CEN: 18	Adult patients required MV in ICU	Age: 62/64; Male (%): 64/36; BMI: 28/27; APACHE II: 17/21	IEN: starting with 80 mL/h, 1 h each time, 4 times a day for 15 days; CEN: 25 mL/h, 24 h a day for 15 days
Beattie et al. (41)	IEN: 11; CEN: 17	Adult ICU patients required gastric enteral nutrition for > 48 h	Age: 60/60; Male (%): 55/59; BMI: 25/24; APACHE II: 13/15	IEN: 200 mL within 0.5–1 h each time, 3 times a day for 2 days; CEN: 24 h a day for 2 days
Goksu and Tuncel (42)	IEN: 31; CEN: 31	Adult intubated patients with sepsis in ICU	Age: 61/61; Male (%): 52/48; BMI: 26/24; APACHE II: 18/18	IEN: 150 mL within 40 min each time, 4 times a day for 7 days; CEN: 24 h a day for 7 days
Hrdy et al. (33)	IEN: 146; CEN: 148	Adult patients required MV for ≥ 72 h in ICU	Age: 62.9/64.4; Male (%): 71/74; BMI: 29.0/29.0; APACHE II: 28/29	IEN: 160–400 mL/h, 0.5–1 h each time, 6 times a day for 5 days; CEN: starting with 25 mL/h, 18 h a day for 5 days
Yao et al. (34)	IEN: 65; CEN: 69	Adult patients in ICU	Age: 64/65; Male (%): 69/80; APACHE II: 16/16	IEN: 2 h each time, 3 times a day for 7 days; CEN: 24 h a day for 7 days
Panwar et al. (32)	IEN: 61; CEN: 59	Adult patients required MV in ICU	Age: 65.1/64.8; Male (%): 67/66; BMI: 27.9/27.2; APACHE II: 22/23	IEN: starting with 150 mL/h, 0.5–1 h each time, 3 times a day for 14 days; CEN: 24 h a day for 14 days
Wilkinson et al. (31)	IEN: 40; CEN: 35	Adult patients required MV for ≥ 48 h in ICU	Age: 55/61; Male (%): 28/26; APACHE II: 21/20	IEN: 60–80 mL a time, 3–5 min each time, 6 times a day for 10 days; CEN: 24 h a day for 10 days
Lee et al. (30)	IEN: 49; CEN: 50	Adult patients required MV in ICU	Age: 66.2/67.5; Male (%): 67.3/66.0; BMI: 22.0/23.3; APACHE II: 27.7/28.6	IEN: starting with 150 mL/h, 1 h each time, 4 times a day for 7 days; CEN: 25 mL/h, 24 h a day for 7 days
Ren et al. (29)	IEN: 32; CEN: 30	Adult patients admitted to ICU and fed through gastric tubes	Age: 66/55; Male (%): 53/63; BMI: 24/23; APACHE II: 19/16	IEN: 2 h each time, 3 times a day for 7 days; CEN: 24 h a day for 7 days
Zhu et al. (28)	IEN: 40; CEN: 38	Adult patients with hemorrhagic stroke, required MV	Age: 59.9/59.6; Male: 55.3/47.5; BMI: 24.6/24.1	IEN: 0.5–1 h each time, 4 times a day for 7 days; CEN: 24 h a day for 7 days
McNelly et al. (27)	IEN: 26; CEN: 59	Adult patients required MV for ≥ 48 h	Age: 55.2/60.3; Male: 66.1/67.8; APACHE II: 23.1/20.2	IEN: 6 times a day for 10 days; CEN: 24 h a day for 10 days
Nasiri et al. (26)	IEN: 20; CEN: 20	Adult patients with sepsis admitted to ICU	Age: 48.3/53.0; Male: 26.5/35.3	IEN: 50–200 mL a time, 15–20 min each time, 6 times a day for 3 days; CEN: 24 h a day for 3 days
Kadamani et al. (25)	IEN: 15; CEN: 15	Adult patients received MV and EN for ≥ 72 h	Age: 61.6/64.7; Male: 66.7/60.0; BMI: 23.3/23.1; APACHE II: 16.0/20.3	IEN: 10–25 min each time, 4–6 times a day for 3 days; CEN: 24 h a day for 3 days
de Tavares Araujo et al. (37)	IEN: 18; CEN: 23	Adult patients in ICU	Age: 68.9/61.3; Male: 55.6/60.9; BMI: 24.6/22.3; APACHE II: 20.7/22.4	IEN: 18 h a day with 6 h night rest for 5 days; CEN: 24 h a day for 5 days
Maurya et al. (35)	IEN: 20; CEN: 20	Adult male patients with head injury requiring MV	Age: 40.2/40.7; Male: 100/100; BMI: 22.0/20.6	IEN: 220 mL a time, 6 times a day; CEN: 18 h a day
MacLeod et al. (24)	IEN: 79; CEN: 81	Adult patients admitted to trauma ICU, required MV ≥ 48 h	Age: 44.6/48.4; Male: 67.1/74.1; APACHE II: 12/14	IEN: 100 mL a time, 30–60 min each time, 6 times a day for 7 days; CEN: 20 mL/h, 24 h a day for 7 days
Chenet et al. (23)	IEN: 56; CEN: 51	Adult patients required MV in ICU	Age: NR; Male: 76.8/76.5	IEN: 125 mL a time, 6 times a day for 7 days; CEN: 25 mL/h, 24 h a day for 7 days
Serpa et al. (22)	IEN: 14; CEN: 14	Adult patients in ICU	Age: 64.9/69.6; Male: 50.0/64.3	IEN: 1 h each time, 6 times a day for 3 days; CEN: 24 h a day for 3 days
Gowardman et al. (40)	IEN: 15; CEN: 12	Adult patients required MV for ≥ 72 h in ICU	Age: 27/30; Male: 87/58; APACHE II: 15/20	IEN: 16 h a day with 8 h night rest for 12 days; CEN: 24 h a day for 12 days
Steevens et al. (21)	IEN: 9; CEN: 9	Adult patients with multiple traumas in ICU	Age: 35.9/37.3; Male: 55.6/77.8; BMI: 27.5/25.4	IEN: 125 mL a time, 15 min each time, 6 times a day for 7 days; CEN: 25 mL/h, 24 h a day for 7 days
Bonten et al. (36)	IEN: 30; CEN: 30	Adult patients required MV in ICU	Age: 68/65; Male: 53.3/63.3; APACHE II: 17/19	IEN: 18 h a day with 6 h night rest for 14 days; CEN: 24 h a day for 14 days
Kocan and Hickisch (39)	IEN: 17; CEN: 17	Adult patients required MV in ICU	NR	IEN: 1 h each time, 6 times a day for 7 days; CEN: 24 h a day for 7 days

IEN: intermittent enteral nutrition; CEN: continuous enteral nutrition; ICU: Intensive Care Unit; MV: mechanical ventilation; NR: not reported; APACHE II: acute physiology and chronic health evaluation II; BMI: body mass index.

### Quality assessment

The methodological quality of included studies was evaluated using the RoB 2 tool (Supplementary Table S3). Overall, 1 study was judged to be at low risk of bias, 18 studies raised some concerns, and 3 studies were at high risk of bias. The most common sources of bias were related to deviations from intended interventions due to the inability to blind participants and personnel to the feeding strategy, and concerns regarding outcome measurement in several studies.

Publication bias was examined using Egger’s regression test combined with funnel plot visualization, which demonstrated no statistically significant asymmetry (Egger’s test, *p* > 0.05 for all outcomes; Supplementary Table S3).

### Outcomes

#### Gastrointestinal complications

**Figure 2:**
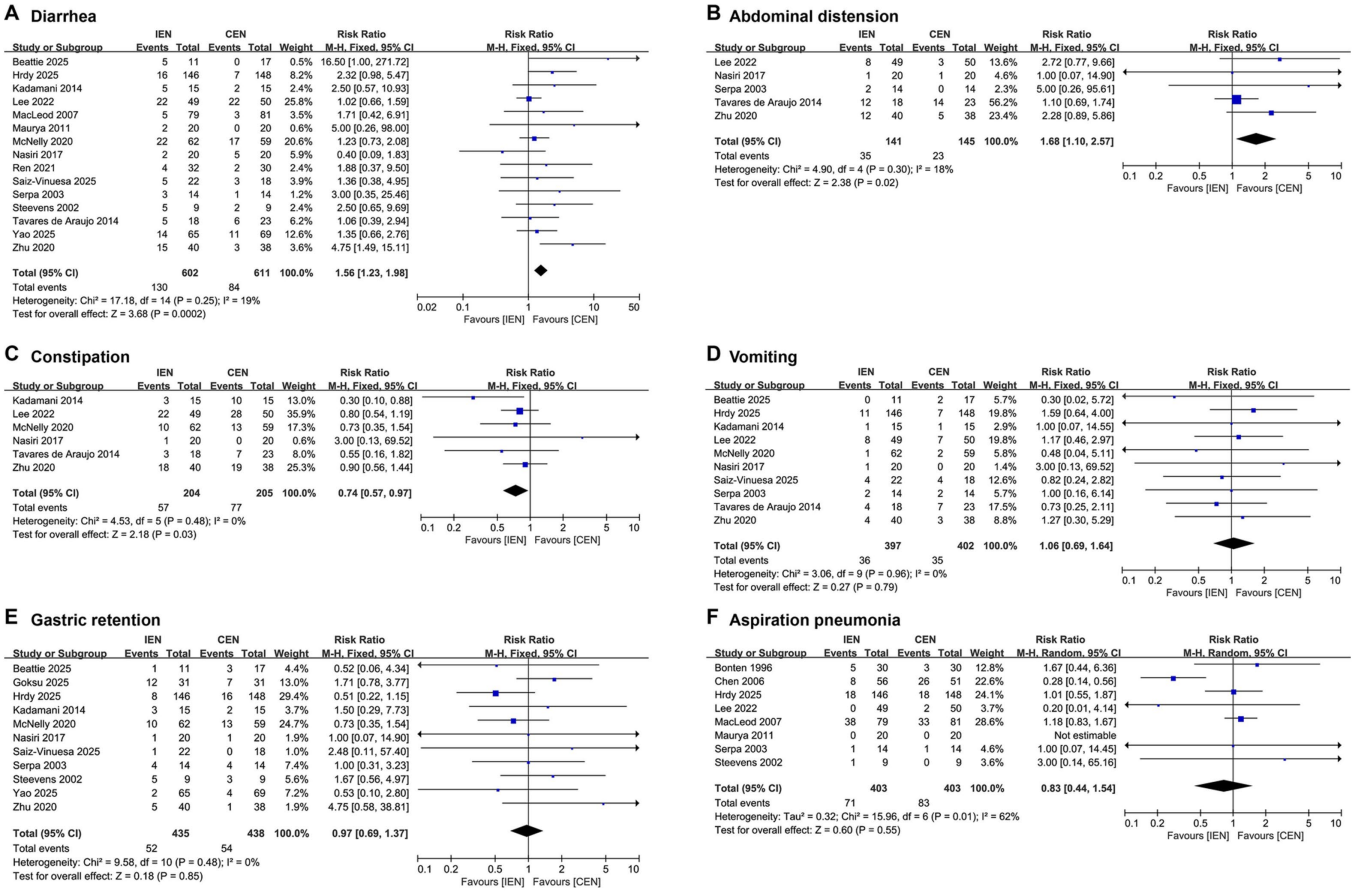
Forest plot comparing the effect of IEN versus CEN on **(A)** diarrhea, **(B)** abdominal distension, **(C)** constipation, **(D)** vomiting, **(E)** gastric retention, **(F)** aspiration pneumonia.

Compared with CEN, IEN was associated with a significantly higher incidence of diarrhea (RR 1.56, 95% CI 1.23–1.98; I^2^ = 19%; Figure 2A) and abdominal distension (RR 1.68, 95% CI 1.10–2.57; I^2^ = 18%; Figure 2B), and a significantly lower incidence of constipation (RR 0.74, 95% CI 0.57–0.97; *I*^2^ = 0%; Figure 2C). No significant differences were identified between IEN and CEN for other gastrointestinal complications: vomiting (RR 1.06, 95% CI 0.69–1.64; *I*^2^ = 0%; Figure 2D), gastric retention (RR 0.97, 95% CI 0.69–1.37; *I*^2^ = 0%; Figure 2E), and aspiration pneumonia (RR 0.92, 95% CI 0.52–1.62; *I*^2^ = 54%; Figure 2F).

In sensitivity analyses, the differences in abdominal distension and constipation between IEN and CEN became statistically non-significant after sequential exclusion of individual studies, indicating potential instability of these findings (Supplementary Table S3).

In the subgroup of patients receiving mechanical ventilation, IEN was associated with significantly higher incidences of diarrhea and abdominal distension and a lower incidence of constipation compared with CEN (Supplementary Table S3). In contrast, no significant differences were observed between the two feeding strategies for any gastrointestinal complication in the non-mechanically ventilated subgroup (Supplementary Table S3).

### Mortality in ICU, length of ICU stay and achievement of nutritional goal

**Figure 3:**
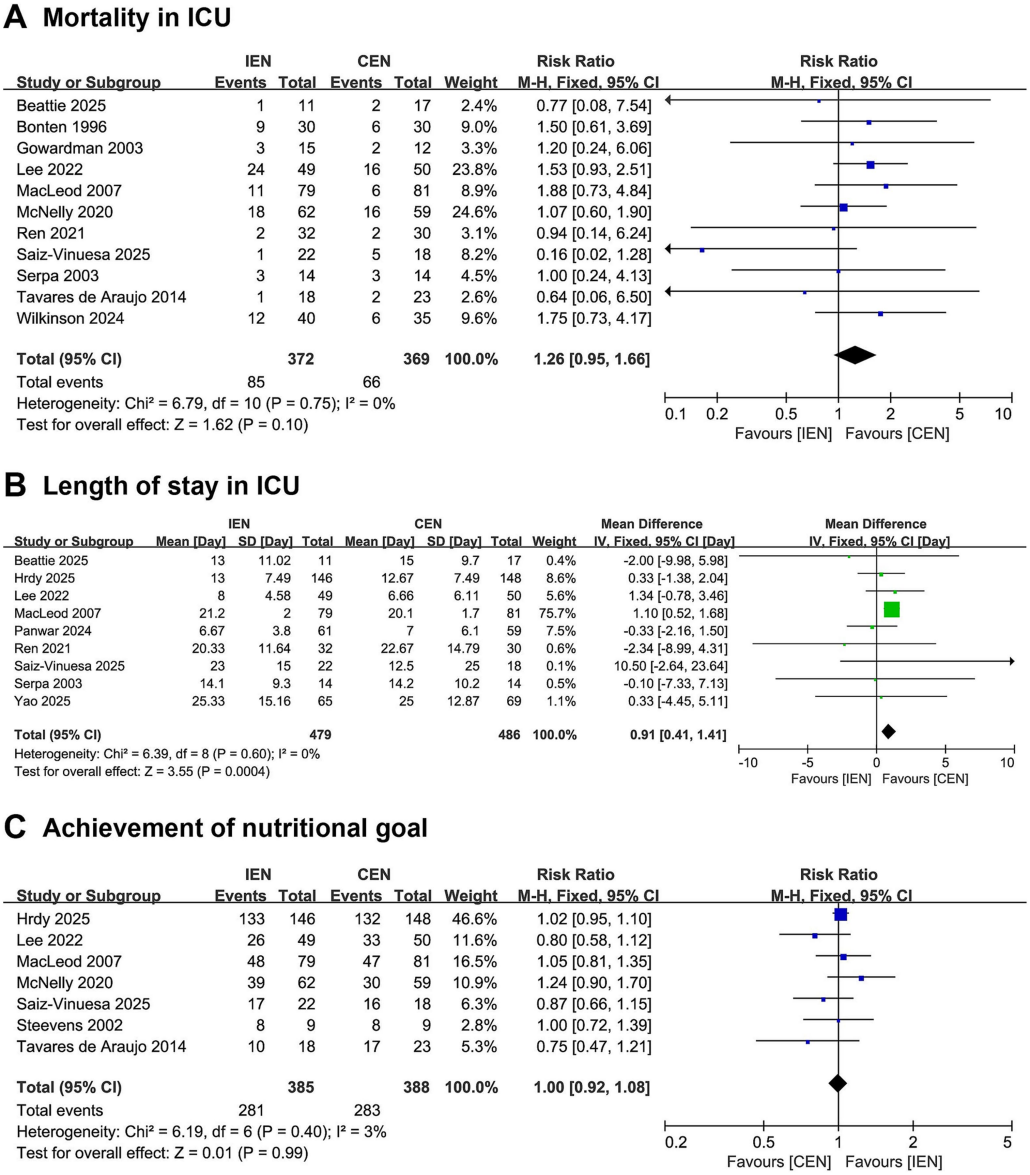
Forest plot comparing the effect of IEN versus CEN on **(A)** mortality in ICU, **(B)** length of stay in ICU, **(C)** achievement of nutritional goal.

Pooled analysis demonstrated a trend toward increased ICU mortality in the IEN group compared with the CEN group; however, this difference did not reach statistical significance (RR 1.26, 95% CI 0.95–1.66; *I*^2^ = 0%; Figure 3A). IEN was associated with significantly prolonged ICU length of stay compared with CEN (MD 0.91 days, 95% CI 0.41–1.41; *I*^2^ = 0%; Figure 3B). No significant difference was observed between the two groups regarding achievement of nutritional goals (RR 1.00, 95% CI 0.92–1.08; *I*^2^ = 3%; Figure 3C).

In sensitivity analysis, the difference in ICU mortality reached statistical significance after excluding the study by Saiz-Vinuesa et al. (38), and the difference in ICU length of stay became non-significant after excluding the study by MacLeod et al. (24), suggesting that specific study characteristics may have influenced these outcomes (Supplementary Table S3).

In the subgroup of mechanically ventilated patients, IEN was associated with significantly longer ICU length of stay (Supplementary Table S3). No statistically significant differences were observed between IEN and CEN for ICU mortality, ICU length of stay, or achievement of nutritional goals in the non-mechanically ventilated subgroup (Supplementary Table S3).

### Post-hoc analysis

**Figure 4:**
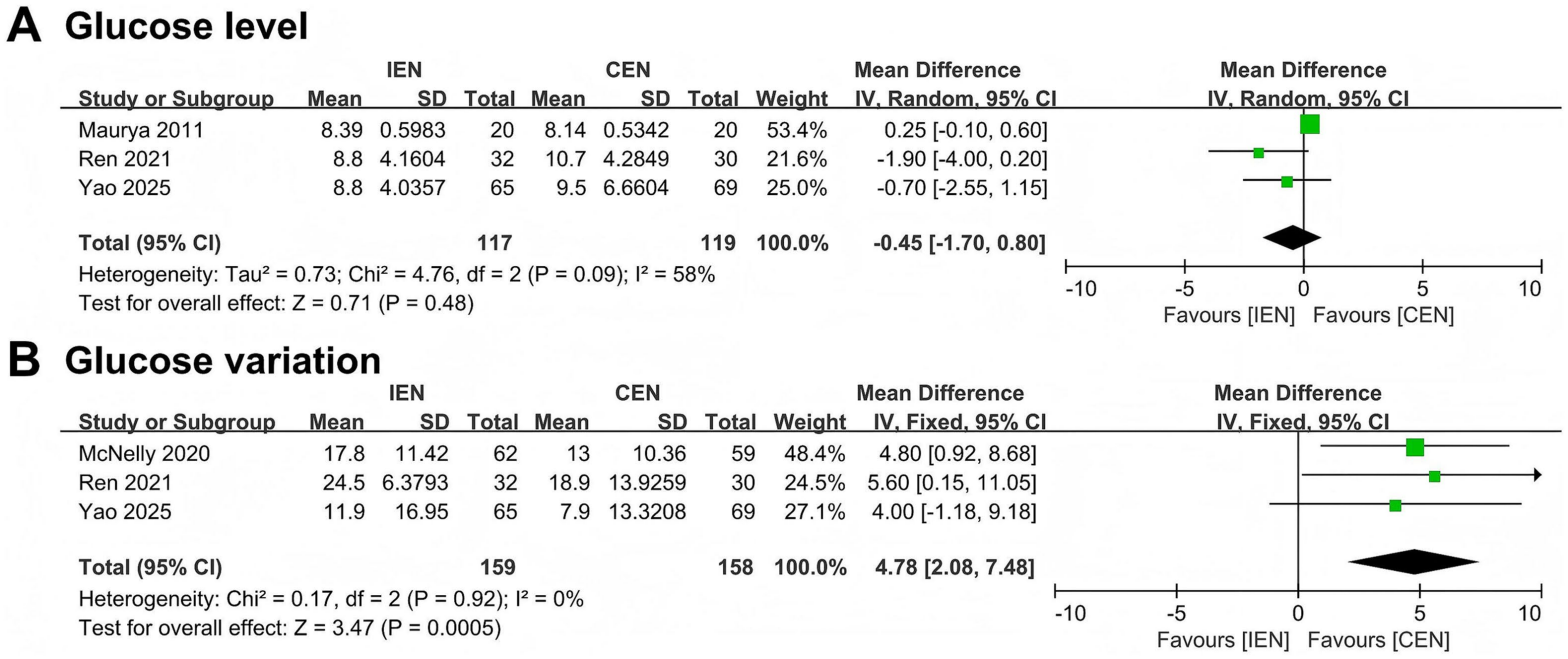
Forest plot comparing the effect of IEN versus CEN on **(A)** glucose level and **(B)** glucose variation.

A *post hoc* analysis was conducted to evaluate the effects of IEN versus CEN on glycemic control parameters (Table 2). Saiz-Vinuesa et al. (38) and Ren et al. (29) reported the incidence of hyperglycemia and hypoglycemia; no significant differences were observed between groups. Data on mean glucose levels and glucose variability were extracted from four RCTs (27, 29, 34, 35). IEN had no significant effect on mean glucose level (MD − 0.45 mmol/L, 95% CI − 1.70 to 0.80; I^2^ = 58%; Figure 4A) but was associated with significantly greater glucose variability compared with CEN (MD 4.78 mg/dL, 95% CI 2.08–7.48; I^2^ = 0%; Figure 4B).

**Table 2:** The effect of intermittent versus continuous enteral nutrition on glucose level and coefficient of glucose variation.

Study	Outcomes
Saiz-Vinuesa et al. (38)	Hyperglycemia: IEN 9.1%, CEN 16.7%, *p* = 0.47 Hypoglycemia: IEN 18.2%, CEN 11.1%, *p* = 0.53
Yao et al. (34)	Glucose level: IEN 8.8 (7.8, 10.8) mmol/L, CEN 9.5 (7.9, 11.1) mmol/L, *p* = 0.391 Coefficient of glucose variation: IEN 11.9 (8.7, 16.1) %, CEN 7.9 (5.6, 11.1) % mmol/L, *p* < 0.001*
Ren et al. (29)	Glucose level: IEN 8.8 (7.3, 10.3) mmol/L, CEN 10.7 (9.1, 12.1) mmol/L, *p* = 0.019* Coefficient of glucose variation: IEN 24.5 (22.2, 27.6) %, CEN 18.9 (13.7, 25.3) % mmol/L, *p* = 0.013* Hyperglycemia: IEN 78.1%, CEN 83.3%, *p* = 0.604
McNelly et al. (27)	Coefficient of glucose variation: IEN 17.8 (14.9, 20.4) %, CEN 13.0 (10.3, 15.7) % mmol/L, *p* < 0.001* Days with hyperglycemia episode: IEN 50.0 (33.3, 72.7) %, CEN 33.3 (18.2, 50.0) % mmol/L, *p* < 0.001*
Maurya et al. (35)	Glucose level: IEN 146–156 mg/dL, CEN 142–151 mg/dL, *p* > 0.05

To address the heterogeneity in IEN delivery protocols, we performed a sensitivity analysis excluding studies that employed cyclic IEN schedules (defined as continuous infusion for ≥ 16 h per day with a nocturnal rest period), as these protocols may overlap with CEN definitions used in other studies. No significant change in the direction of results in the sensitivity analysis that omit studies that employed cyclic IEN schedules, indicating the good robustness (Supplementary Table S3).

Additionally, to address potential changes in clinical practices over time, we further conducted a post-hoc temporal subgroup analysis comparing trials published from 2020 through 2025 with trials published before 2020. In the 2020–2025 subgroup, IEN was associated with increased diarrhea and increased abdominal distension compared with CEN. Conversely, in the pre-2020 subgroup, IEN was associated with a decreased incidence of constipation and prolonged ICU length of stay compared with CEN. For other outcomes, no statistically significant differences were observed between IEN and CEN across the temporal subgroups. These findings suggest that the balance between gastrointestinal tolerance and clinical outcomes may differ across periods, potentially reflecting evolving ICU practices and patient management strategies.

## Discussion

### Key findings

In this updated meta-analysis of 22 RCTs comparing IEN with CEN in critically ill patients, we found that IEN was associated with higher rates of diarrhea and abdominal distension, longer ICU length of stay, and a lower incidence of constipation compared with CEN. Notably, these effects were predominantly observed in patients receiving mechanical ventilation, with no significant differences detected in the non-ventilated subgroup. These findings extend prior work and provide a more precise characterization of the gastrointestinal tolerance trade-offs between the two feeding strategies in the ICU setting.

### Clinical implications and mechanistic considerations

The findings of this meta-analysis have important implications for nutritional support strategies in critically ill patients and warrant careful consideration of the physiological mechanisms underlying the observed differences. It is well established that a substantial proportion (>40%) of critically ill patients receiving enteral nutrition experience gastrointestinal complications, which represent common clinical challenges in this population (43). Although our pooled results indicate that IEN is associated with greater gastrointestinal intolerance and longer ICU length of stay compared with CEN, the mechanisms underlying these observations are likely multifactorial.

First, gastrointestinal motility is frequently impaired in critical illness. Delayed gastric emptying and broader dysmotility are common consequences of sepsis, sedation, vasopressor therapy, and mechanical ventilation; these abnormalities reduce the capacity of the stomach and small intestine to accommodate episodic loads of enteral formula. Patients with persistent motility dysfunction may therefore tolerate slow, continuous delivery better than intermittent boluses, which transiently increase gastric volume and intraluminal pressure and can precipitate feeding intolerance (44).

Subgroup analysis indicates that the clinical outcomes of IEN versus CEN are significantly modified by mechanical ventilation status. Mechanically ventilated patients exhibited more pronounced differences between the two feeding strategies, likely due to the compounded gastrointestinal dysfunction associated with critical illness and iatrogenic factors such as sedatives and neuromuscular blocking agents (45, 46). These factors can significantly delay gastric emptying and impair intestinal motility, potentially exacerbating feeding intolerance with bolus delivery methods. This observed discrepancy underscores the necessity for an individualized nutritional approach tailored to patient-specific factors, including baseline gastrointestinal function, level of sedation, and ventilator dependence. Importantly, illness severity likely represents a critical effect modifier. Although APACHE II scores were reported in a subset of included studies, incomplete reporting precluded a formal severity-adjusted meta-analysis. The observed differences between mechanically ventilated and non-ventilated patients may partially reflect underlying differences in disease severity rather than the feeding strategy alone.

Second, intermittent feeding produces cyclical postprandial surges in gastric and intestinal content that can increase the risk of gastroesophageal reflux and aspiration in patients with impaired lower esophageal sphincter competence or delayed gastric emptying. Several clinical reports and reviews have highlighted a signal for increased regurgitation and aspiration with intermittent or bolus delivery in vulnerable ICU populations, and this mechanism provides a plausible explanation for worse pulmonary or global outcomes when bolus feeding is employed in patients with persistent gastrointestinal dysfunction (47, 48).

Third, the *post hoc* analysis demonstrated that IEN significantly increases glucose variability despite achieving comparable mean blood glucose levels to CEN. Intermittent delivery of substantial carbohydrate loads generates postprandial hyperglycemic peaks, whereas interprandial fasting periods predispose to relative hypoglycemia. This oscillating pattern contrasts with the steady-state glucose homeostasis achievable with CEN, which provides stable nutrient delivery and facilitates tighter glycemic control (49). Emerging evidence suggests that glycemic variability may be independently associated with adverse outcomes in critically ill patients (50, 51), representing a potential mechanism through which IEN may contribute to increased mortality and prolonged ICU length of stay.

Finally, it is important to acknowledge that continuous feeding is not intrinsically physiological and may have its own limitations, including suppression of fasting-related motility patterns and increased risk of constipation. Consequently, the apparent benefit of CEN in many ICU trials may reflect a pragmatic advantage in the presence of persistent gastrointestinal dysfunction rather than indicating that continuous delivery restores normal gastrointestinal physiology (52, 53). This interpretation is consistent with ASPEN guidelines (9), which tend to favor continuous delivery in critically ill populations at high risk for intolerance while also supporting targeted strategies (prokinetic agents, post-pyloric feeding, volume or formula adjustments) for patients in whom bolus or cyclic feeding is being considered.

In summary, our findings support a patient-centered approach: in patients with evidence of persistent gastrointestinal dysfunction, CEN may reduce intolerance and downstream complications, whereas intermittent feeding could be evaluated in stable patients with preserved motility.

In addition to the mechanical ventilation subgroup analysis, we performed a temporal subgroup analysis restricted to RCTs published between 2020 and 2025 to account for potential changes in ICU practice and patient management over time. In these more recent trials, IEN demonstrated a pattern of worse gastrointestinal tolerance, specifically increasing diarrhea and abdominal distension compared with CEN. In contrast, in trials published before 2020, IEN showed different associations, namely reduced constipation and increased length of ICU stay. Taken together, these temporal differences indicate that the magnitude and direction of specific clinical effects may vary with contemporary ICU protocols, including changes in sedation/ventilation strategies, feeding advancement practices, and supportive care. Therefore, clinicians should interpret the overall pooled effects with consideration of both patient characteristics and the likely evolution of practice patterns across study eras.

### Relationship to other studies

The systematic review conducted by Ma et al. (48), which analyzed 14 RCTs published before 2020, identified elevated feeding intolerance rates associated with bolus administration, yet detected no meaningful disparities in additional clinical metrics. Beyond the temporal limitations of that evidence synthesis, the predominant inclusion of Chinese-language trials may limit the generalizability of their conclusions to diverse international populations.

In a subsequent investigation, Heffernan and colleagues performed an evidence synthesis encompassing 14 English-language RCTs, demonstrating comparable outcomes between feeding modalities regarding mortality, diarrhea, elevated gastric residual volumes, pneumonia, and microbial colonization (14). A further meta-analysis by Qu et al. (12) reported associations between bolus enteral feeding and increased frequencies of diarrhea and abdominal distension, together with prolonged ICU stay, while simultaneously observing reduced constipation prevalence.

A principal contribution of our investigation lies in establishing the association between IEN and prolonged ICU length of stay relative to continuous delivery methods. This observation represents a conceptual advance from previous meta-analyses that predominantly emphasized gastrointestinal tolerance and adverse events with insufficient consideration of definitive clinical endpoints. Moreover, our stratified analyses demonstrate that the adverse consequences of bolus feeding manifest primarily among patients requiring mechanical ventilation, whereas no substantial differences emerge in spontaneously breathing populations.

Recently, Panwar et al. (15) evaluated continuous versus bolus nutritional delivery specifically among mechanically ventilated patients. Their pooled analysis of 8 RCTs failed to identify significant differences in critical clinical outcomes (including mortality, ICU length of stay, gastrointestinal intolerance, and pneumonia) between nutritional strategies (15). Through incorporation of contemporary trial evidence, our meta-analysis elucidates clinically relevant distinctions that have received inadequate attention in prior literature.

### Implications for practice and research

Based on the present analysis, CEN emerges as the preferable feeding strategy for critically ill adults, particularly those receiving mechanical ventilation. The evidence indicates that CEN is associated with improved gastrointestinal tolerance and reduced ICU length of stay. For constipation associated with CEN, implementation of targeted interventions, such as prokinetic agents or fiber supplementation, is recommended rather than switching to IEN, given the potential risks associated with intermittent feeding. In clinical decision-making, practitioners should perform an individualized risk–benefit assessment when selecting a feeding modality, incorporating patient-specific factors including baseline gastrointestinal function, illness severity, and evolving tolerance patterns.

Future investigations should prioritize large-scale RCTs designed to identify patient subgroups that might benefit from IEN. For example, patients with preserved gut function or those in the convalescent phase of illness. Additionally, examining long-term outcomes is essential to assess whether any mortality differences observed in the ICU translate to improved hospital survival and functional recovery. Further research incorporating health economic evaluations would be valuable to determine the cost-effectiveness of different feeding strategies, accounting for direct costs and healthcare resource utilization.

Importantly, several large-scale, multicenter RCTs are currently underway to more rigorously evaluate the clinical efficacy and safety of different EN strategies in critically ill populations. The DC-SCENIC trial (54) plans to enroll more than 300 participants and maintains rigorous control over caloric and protein intake across study arms. The DINE-Normal trial (55) investigates the effects of IEN on physiological, hormonal, and metabolic responses in critically ill adults. The ENNUT trial (54) enrolls critically ill adults requiring mechanical ventilation and vasopressor support to evaluate the comparative effects of IEN and CEN.

### Limitations

Several limitations of this meta-analysis should be acknowledged. First, although 22 RCTs were included, the sample sizes of individual studies were generally small, and methodological quality varied, which may have affected the robustness of pooled estimates (56). Second, blinding of clinicians to the feeding strategy was rarely feasible, potentially affecting subjective gastrointestinal endpoints. Consequently, most included RCTs were characterized by moderate-to-high risk of bias. Third, heterogeneity in formulas (energy density, fiber content), feeding routes (gastric versus post-pyloric), residual volume thresholds, and bowel management protocols could influence intolerance profiles and limit generalizability. The lack of reported data on energy and protein intake prevents us from excluding these factors as potential contributors to the observed effects. Fourth, several gastrointestinal outcomes (including abdominal distension, constipation, and gastric retention) were assessed subjectively without standardized diagnostic criteria, potentially affecting the accuracy of outcome assessment. Fifth, an important methodological consideration is the substantial heterogeneity in IEN delivery protocols across included studies. Feeding frequency ranged from multiple daily boluses to extended infusion schedules approaching 18 h per day. Such regimens may partially overlap with continuous feeding strategies, thereby diluting the contrast between intervention groups. Although sensitivity analyses excluding these studies yielded consistent results, this variability remains a key limitation and may have attenuated the true effect size.

Finally, the included studies span nearly four decades, during which substantial advancements in critical care including ventilatory support, glycemic control, and nutritional practices have occurred. Although our temporal subgroup analysis of studies published from 2020 to 2025 revealed outcome-specific differences, temporal heterogeneity may still influence the generalizability of pooled estimates.

## Conclusion

This updated meta-analysis of 22 RCTs comparing IEN and CEN in critically ill patients demonstrates that the two feeding strategies exert differential effects on gastrointestinal tolerance and clinical outcomes. Compared with CEN, IEN is associated with higher rates of diarrhea and abdominal distension and prolonged ICU length of stay, although it reduces the incidence of constipation. These findings are particularly relevant for patients receiving mechanical ventilation, who appear to be at greatest risk for complications with intermittent feeding. Based on current evidence, CEN is the preferable feeding strategy for most critically ill patients, although individualized approaches considering patient-specific factors remain essential. Further high-quality, large-scale randomized trials are warranted to confirm these findings and to identify patient subgroups who might benefit from intermittent feeding strategies.

## Data Availability

The original contributions presented in the study are included in the article/Supplementary material, further inquiries can be directed to the corresponding author.

## Footnotes

1
https://osf.io/krs8v
