# Supplementary material for: Intermittent versus continuous enteral nutrition in critically ill patients: an updated systematic review and meta-analysis of randomized controlled trials
Source: Front Nutr. 2026 Apr 21;13:1786580. doi: 10.3389/fnut.2026.1786580 (PMC13139002; doi:10.3389/fnut.2026.1786580)
Supplement: Supplementary file 2 [file Table_2.docx]

**Supplementary Material 2: Searching strategies**

**Pubmed**

#1 Intensive Care Units [MeSH Terms] OR Critical Care [MeSH Terms] OR Critical Illness [MeSH Terms] OR icu [Title/Abstract] OR Critical Care [Title/Abstract] OR critically ill [Title/Abstract] OR Intensive Care [Title/Abstract]

#2 Continuous [Title/Abstract]

#3 Intermittent [Title/Abstract] OR Bolus [Title/Abstract] OR sequential [Title/Abstract]

#4 feed [Title/Abstract] OR feeding [Title/Abstract] OR enteral [Title/Abstract]

#5 randomized controlled trial [MeSH Terms] OR randomized [Title/Abstract] OR randomised [Title/Abstract]

#1 AND #2 AND #3 AND #4 AND #5

**Embase**

#1 ‘Intensive Care Units’:ti,ab,kw OR ‘Critical Care’:ti,ab,kw OR ' intensive care unit '/exp OR ' Intensive Care '/exp OR ‘Critical Illness’:ti,ab,kw OR ‘critically ill’:ti,ab,kw OR ‘Intensive Care’:ti,ab,kw OR ‘ICU’:ti,ab,kw

#2 ' Continuous ':ti,ab,kw

#3 ' Intermittent ':ti,ab,kw OR ' Bolus '/exp OR ' sequential '/exp

#4 ' feed ':ti,ab,kw OR ' feeding '/exp OR ' enteral '/exp

#5 'randomized controlled trial'/de OR 'randomized controlled trial'/exp

#1 AND #2 AND #3 AND #4 AND #5

**Scopus**

#1 TITLE-ABS-KEY (Intensive Care Units) OR TITLE-ABS-KEY (Critical Care) OR TITLE-ABS-KEY (Critical Illness) OR TITLE-ABS-KEY (icu) OR TITLE-ABS-KEY (critically ill) OR TITLE-ABS-KEY (Intensive Care)

#2 TITLE-ABS-KEY (Continuous)

#3 TITLE-ABS-KEY (Intermittent) OR TITLE-ABS-KEY (Bolus) OR TITLE-ABS-KEY (sequential)

#4 TITLE-ABS-KEY (feed) OR TITLE-ABS-KEY (feeding) OR TITLE-ABS-KEY (enteral)

#5 TITLE-ABS-KEY (randomized) OR TITLE-ABS-KEY (random) OR TITLE-ABS-KEY (randomised)

#1 AND #2 AND #3 AND #4 AND #5

**Cochrane Library**

#1 (Intensive Care Units):ti,ab,kw OR (Critical Care):ti,ab,kw OR (Critical Illness):ti,ab,kw OR (icu):ti,ab,kw OR (critically ill):ti,ab,kw OR (Intensive Care):ti,ab,kw

#2 (Continuous):ti,ab,kw

#3 (Intermittent):ti,ab,kw OR (Bolus):ti,ab,kw OR (sequential):ti,ab,kw

#4 (feed):ti,ab,kw OR (feeding):ti,ab,kw OR (enteral):ti,ab,kw

#5 (randomized):ti,ab,kw OR (randomised):ti,ab,kw OR (random):ti,ab,kw

#1 AND #2 AND #3 AND #4 AND #5
