# Supplementary material for: Intermittent versus continuous enteral nutrition in critically ill patients: an updated systematic review and meta-analysis of randomized controlled trials
Source: Front Nutr. 2026 Apr 21;13:1786580. doi: 10.3389/fnut.2026.1786580 (PMC13139002; doi:10.3389/fnut.2026.1786580)
Supplement: Supplementary file 3 [file Table_3.docx]

**Supplementary Material 3:** Risk of bias 2 of all included studies, publication bias assessment by funnel plot and Egger’s test, forest plots for sensitivity analyses and subgroup analysis


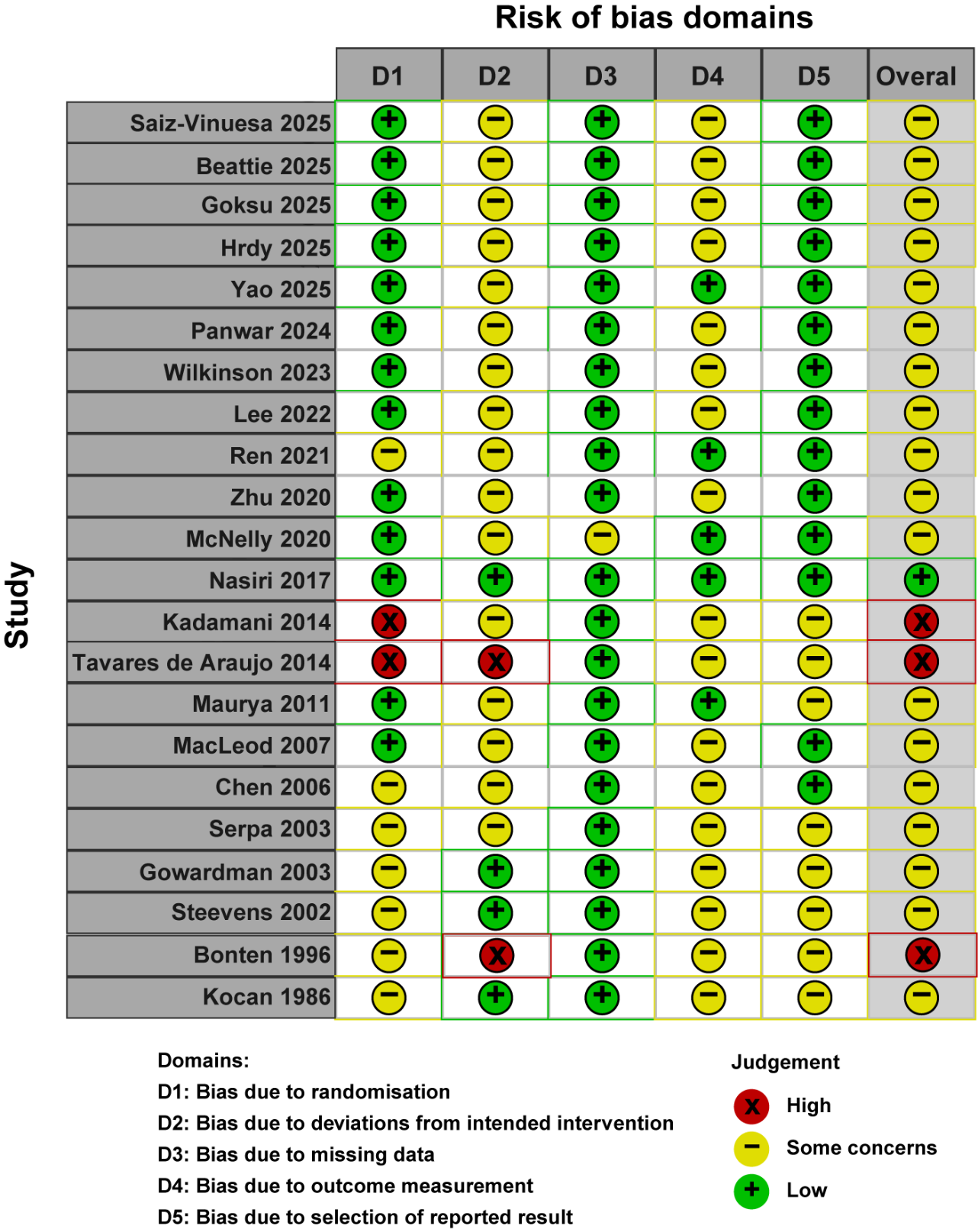


Figure 1: Risk of bias 2 of all included studies


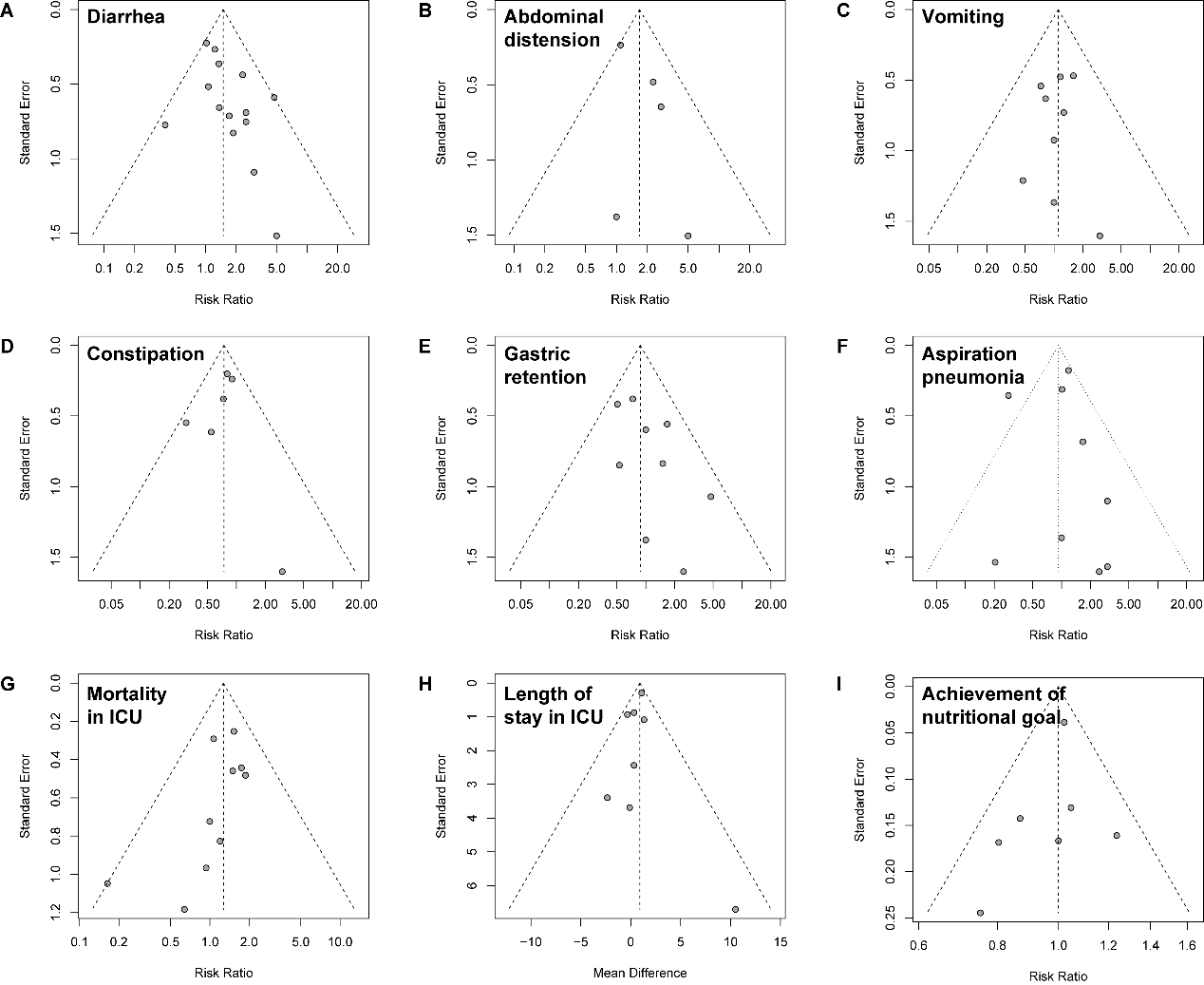


Figure 2: Publication bias assessment by funnel plot and Egger’s test (A) diarrhea, Egger’s test P=0.0687, (B) abdominal distension, Egger’s test P=0.2128, (C) vomiting, Egger’s test P=0.7833, (D) constipation, Egger’s test P=0.6171, (E) gastric retention, Egger’s test P=0.1017, (F) aspiration pneumonia, Egger’s test P=0.9611, (G) mortality, Egger’s test P=0.1288, (H) length of stay in ICU, Egger’s test P=0.6097, (I) achievement of nutritional goal, Egger’s test P=0.3706


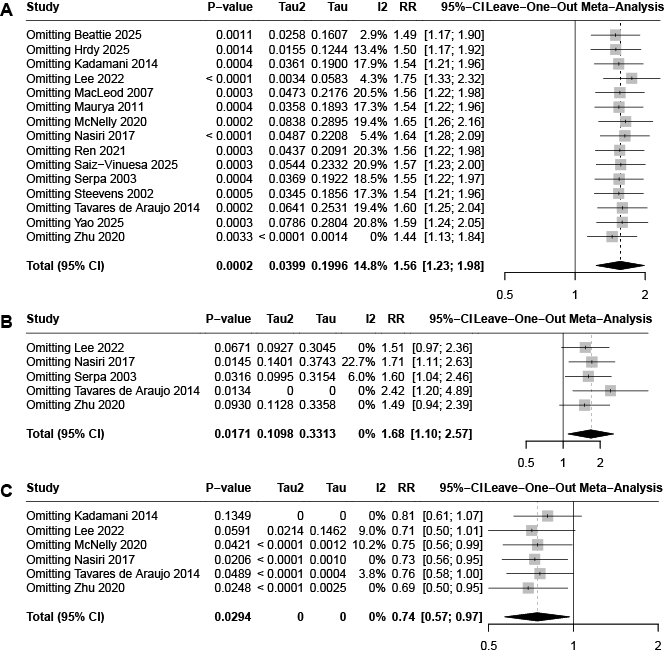


Figure 3: Forest plots for sensitivity analyses, (A) diarrhea, (B) abdominal distension, (C) vomiting


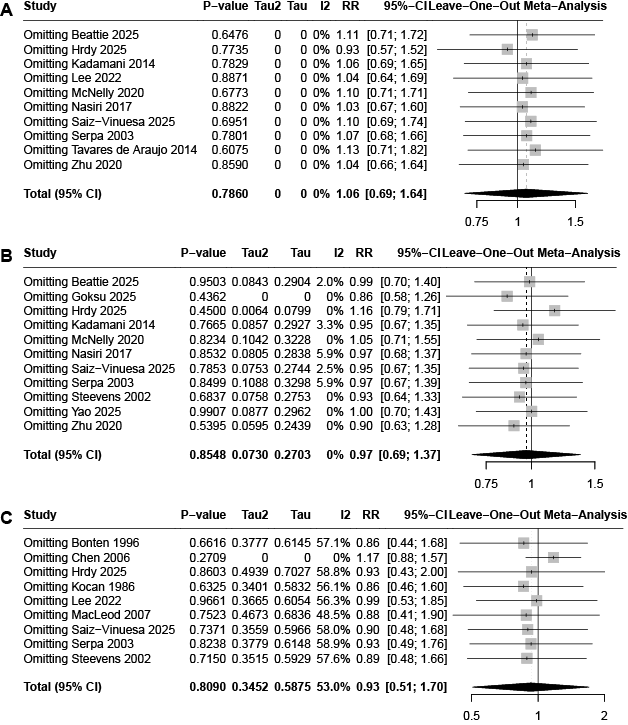


Figure 4: Forest plots for sensitivity analyses, (A) constipation, (B) gastric retention, (C) aspiration pneumonia


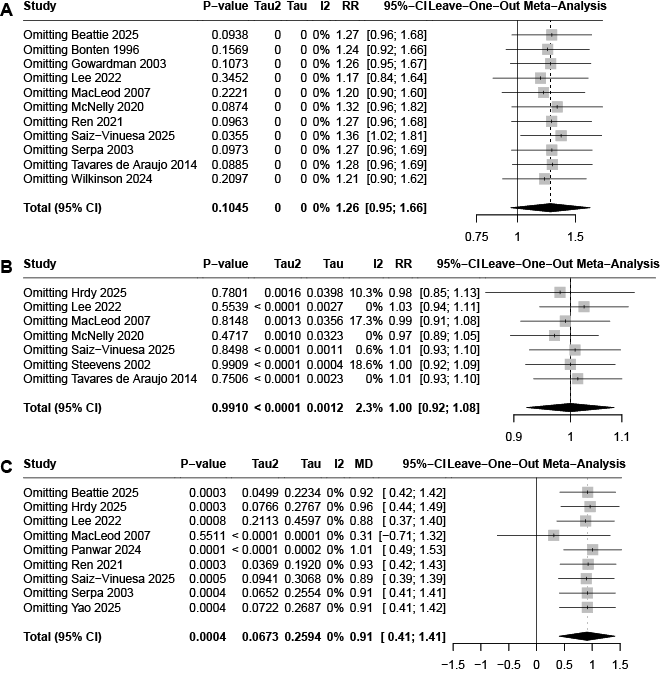


Figure 5: Forest plots for sensitivity analyses, (A) mortality in ICU, (B) length of stay in ICU, (C) achievement of nutritional goal


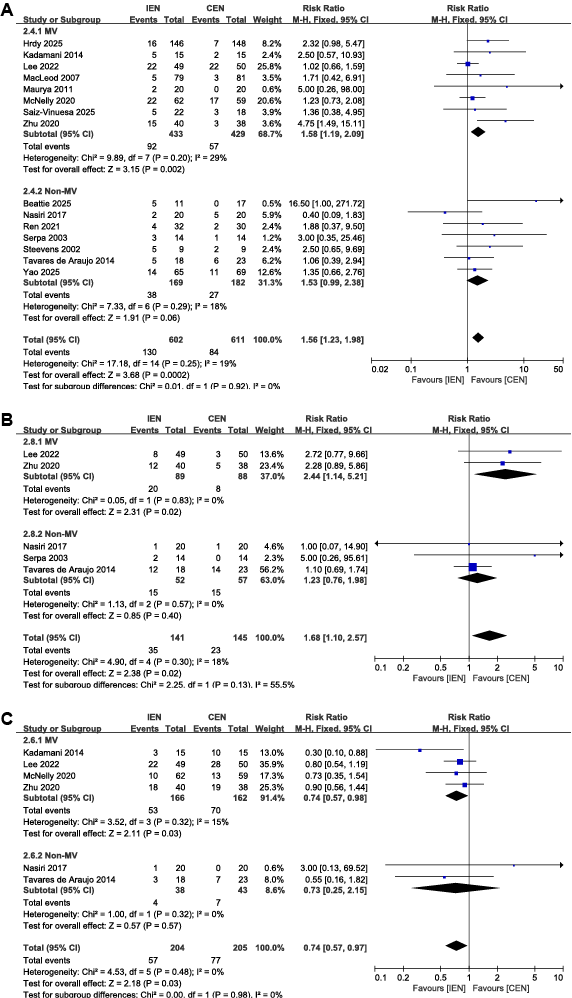


Figure 6: Subgroup analysis stratified by MV subgroup and non-MV subgroup for (A) diarrhea, (B) abdominal distension, (C) constipation


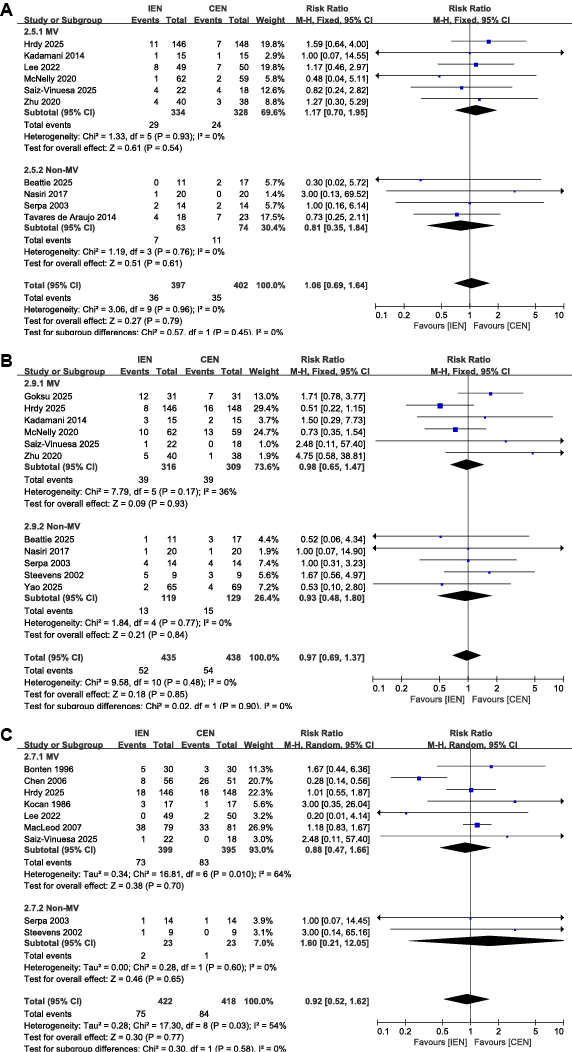


Figure 7: Subgroup analysis stratified by MV subgroup and non-MV subgroup for (A) vomiting, (B) gastric retention, (C) aspiration pneumonia


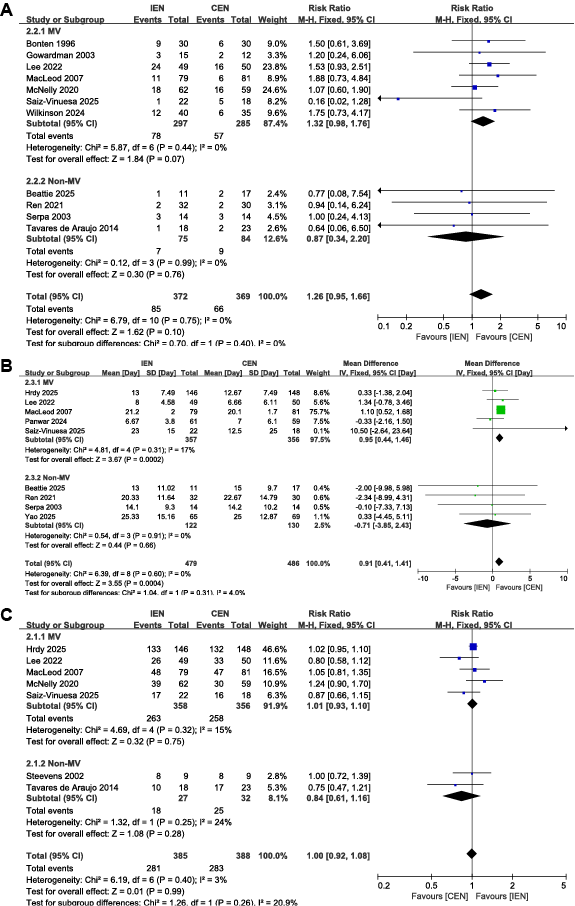


Figure 8: Subgroup analysis stratified by MV subgroup and non-MV subgroup for (A) mortality in ICU, (B) length of ICU stay, (C) achievement of nutritional goal


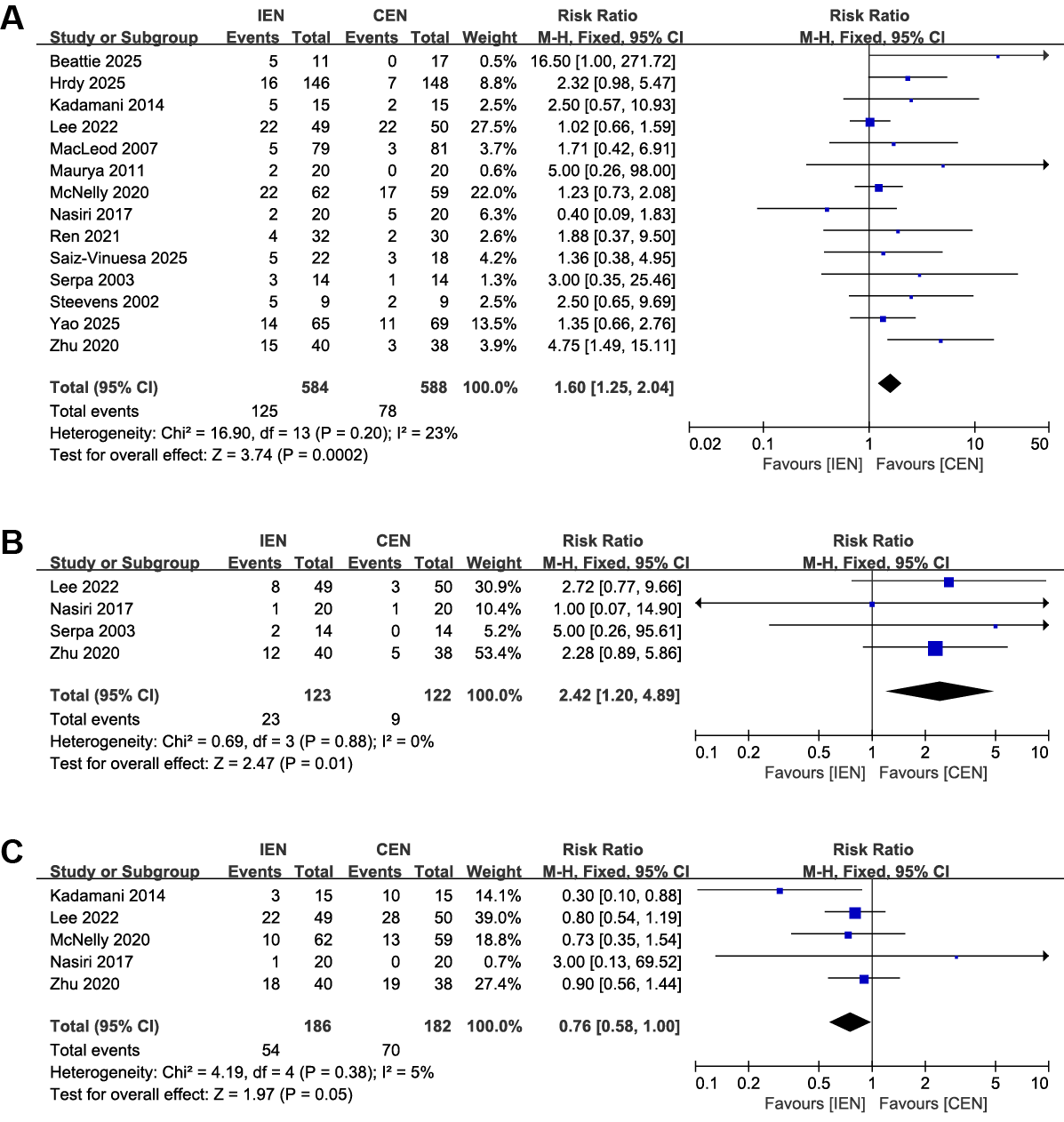


Figure 9: Sensitivity analysis excluding studies that employed cyclic IEN schedules for (A) diarrhea, (B) abdominal distension, (C) constipation


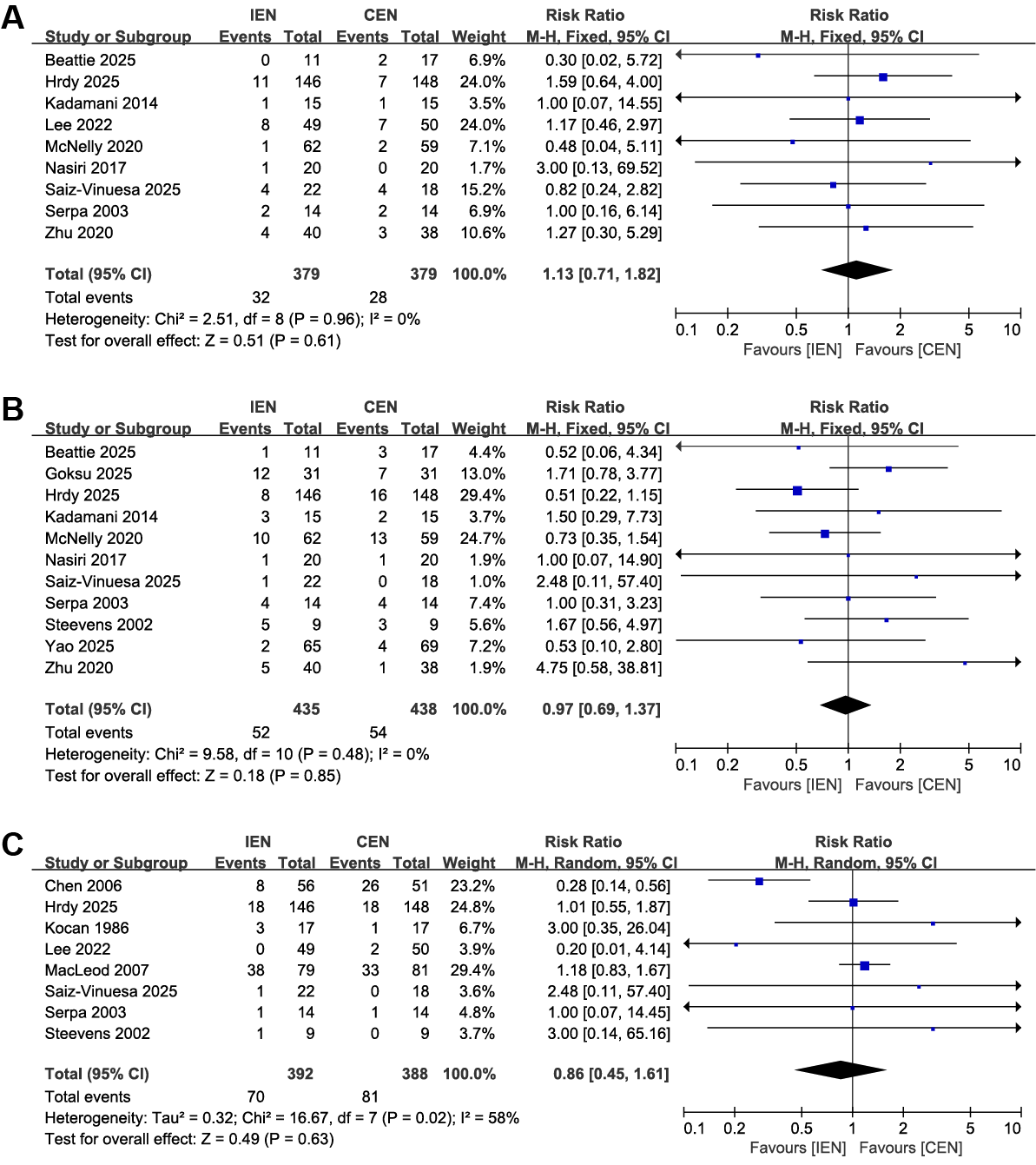


Figure 10: Sensitivity analysis excluding studies that employed cyclic IEN schedules for (A) vomiting, (B) gastric retention, (C) aspiration pneumonia


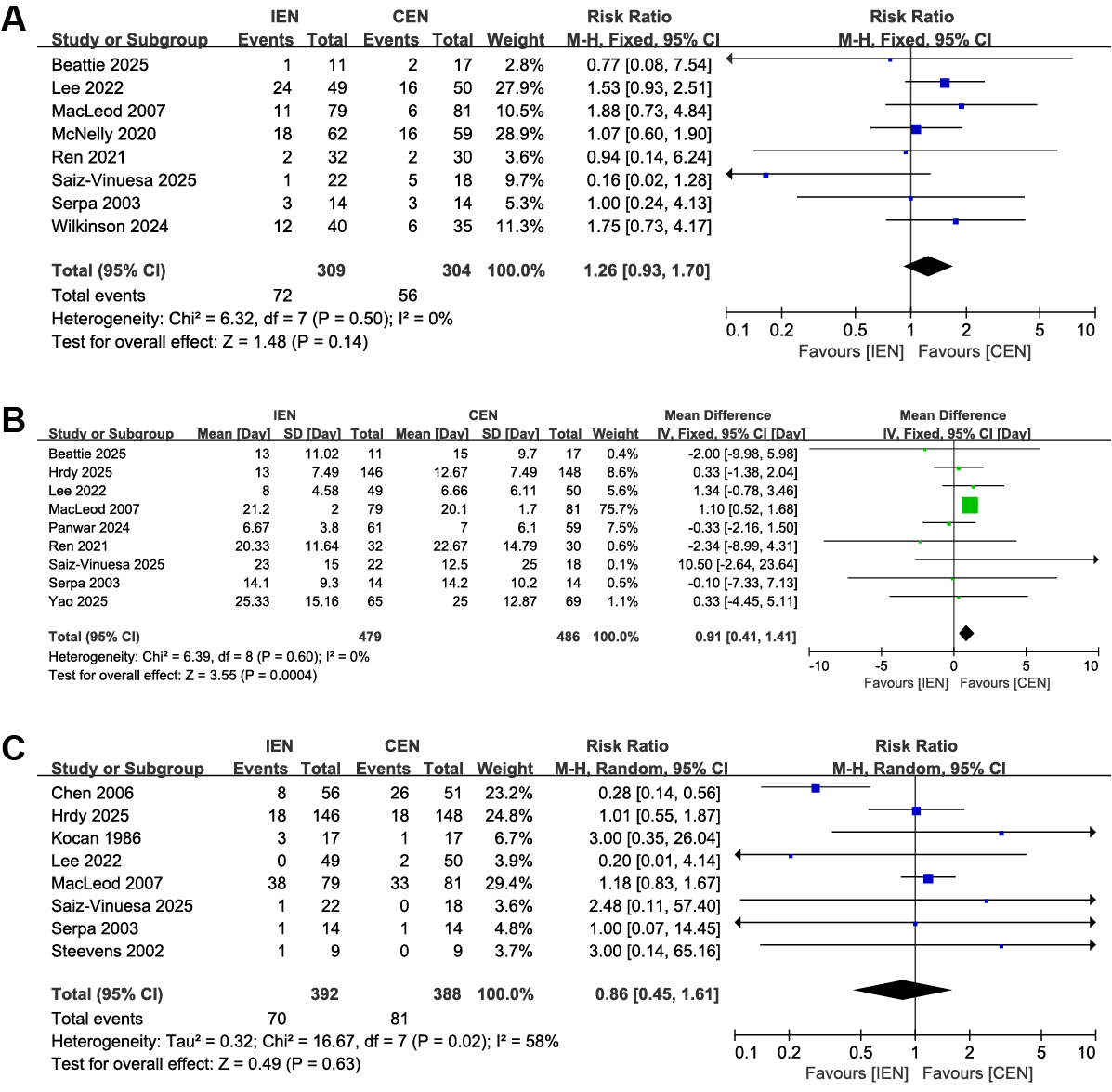


Figure 11: Sensitivity analysis excluding studies that employed cyclic IEN schedules for (A) mortality in ICU, (B) length of ICU stay, (C) achievement of nutritional goal


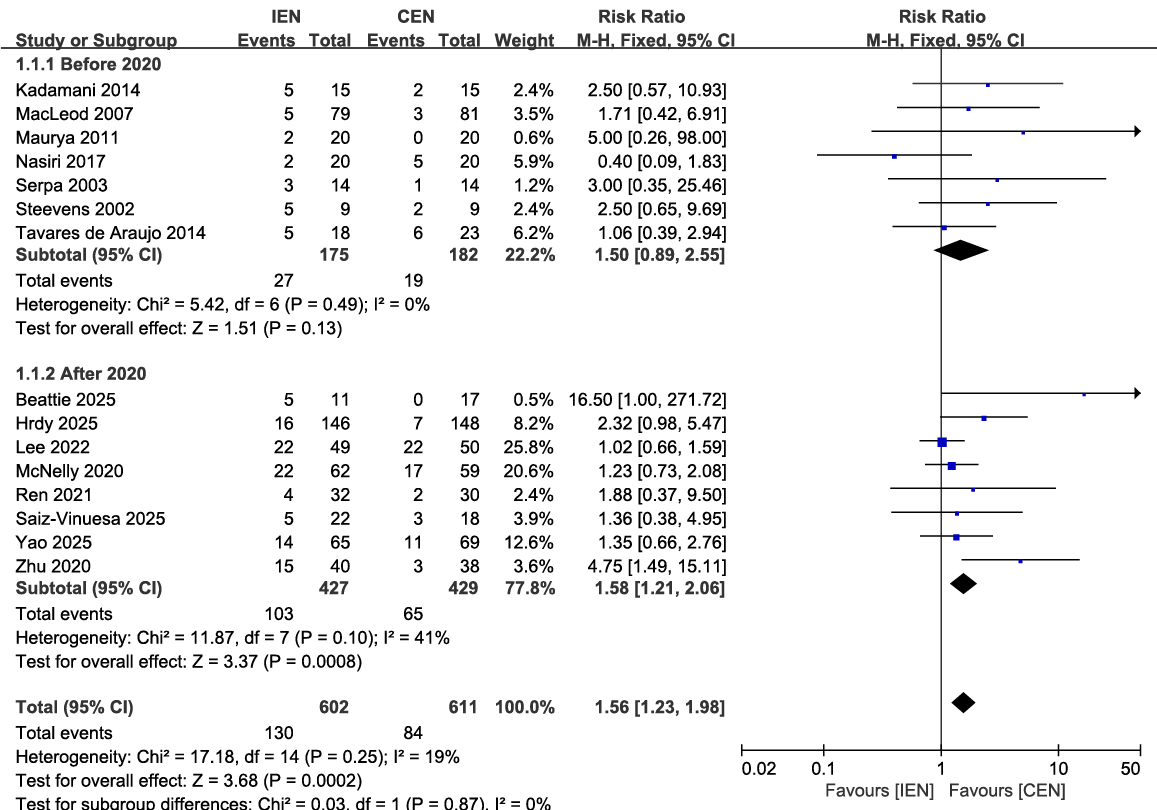


Figure 12: Subgroup analysis stratified by publication time for diarrhea


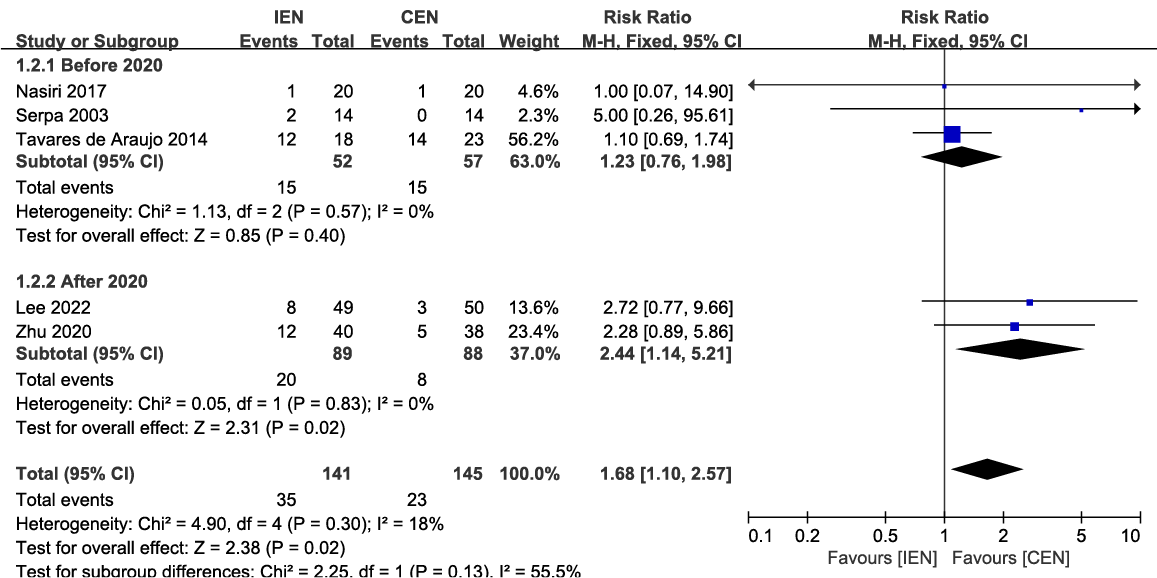


Figure 13: Subgroup analysis stratified by publication time for abdominal distension


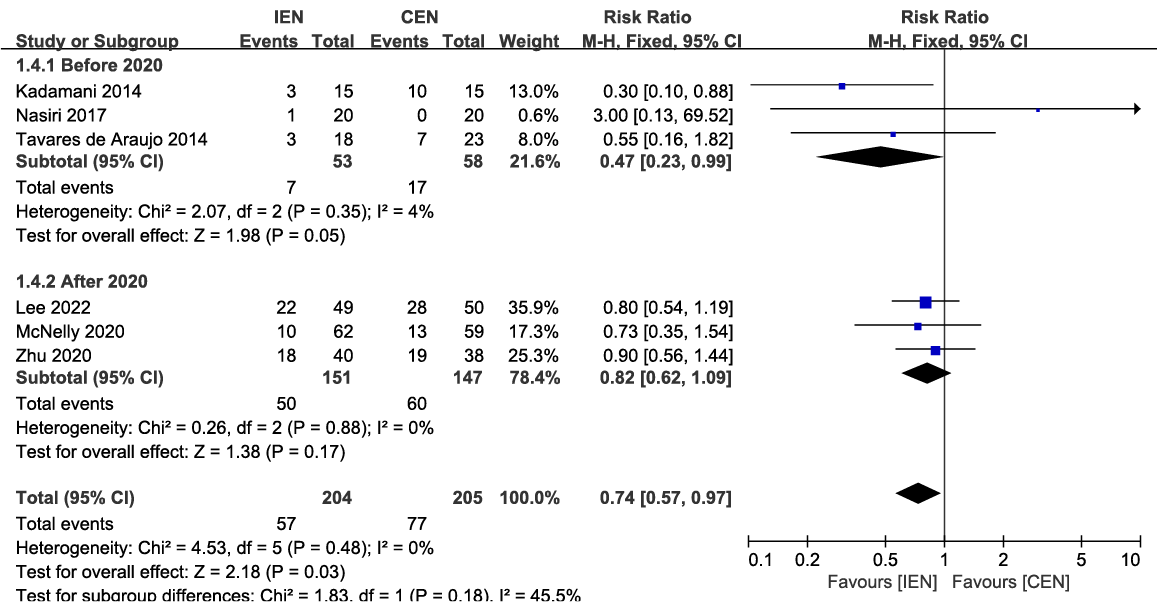


Figure 14: Subgroup analysis stratified by publication time for constipation


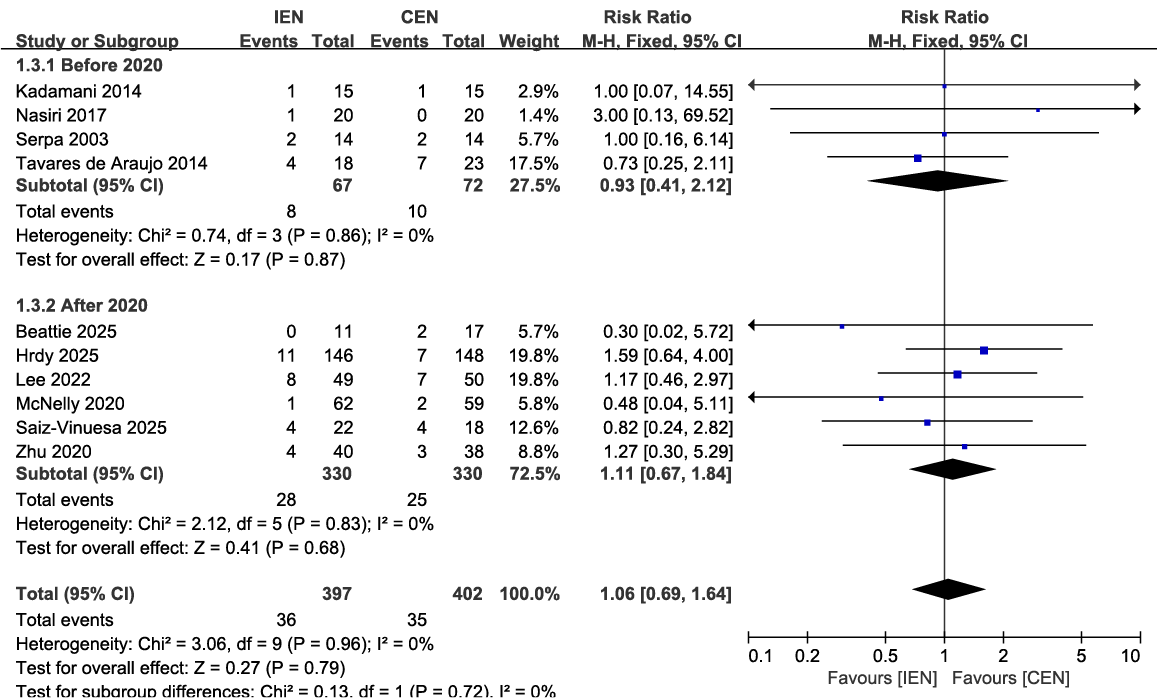


Figure 15: Subgroup analysis stratified by publication time for vomiting


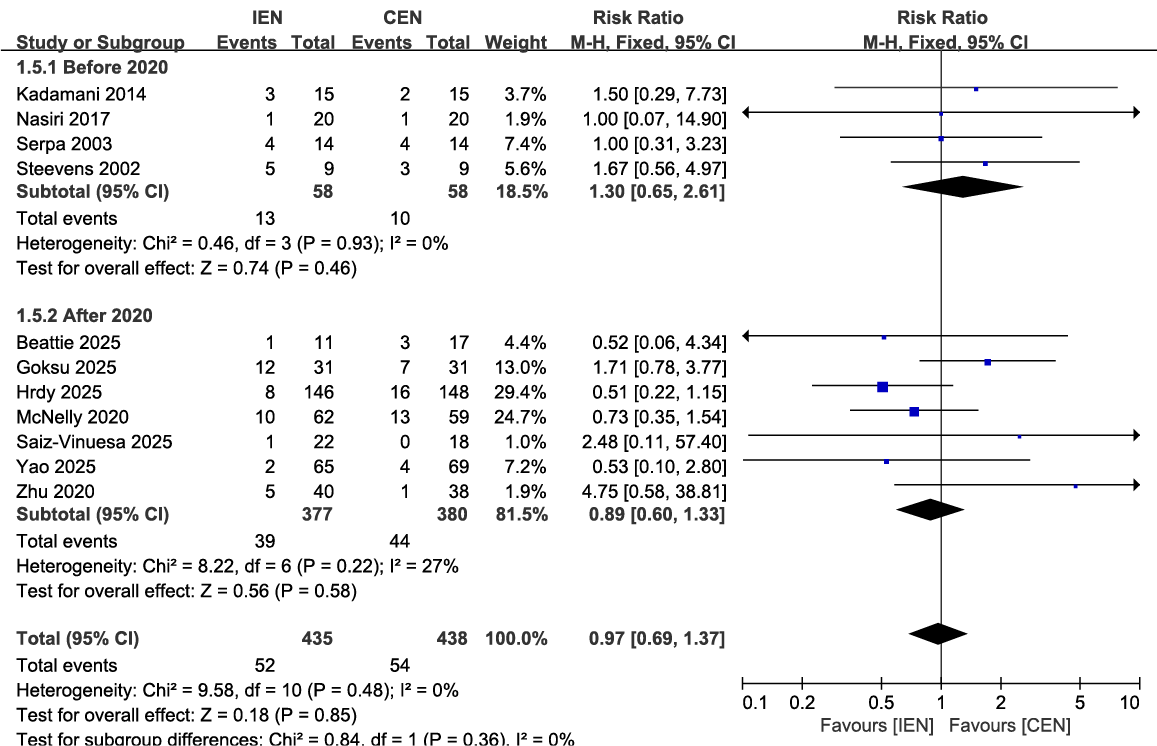


Figure 16: Subgroup analysis stratified by publication time for gastric retention


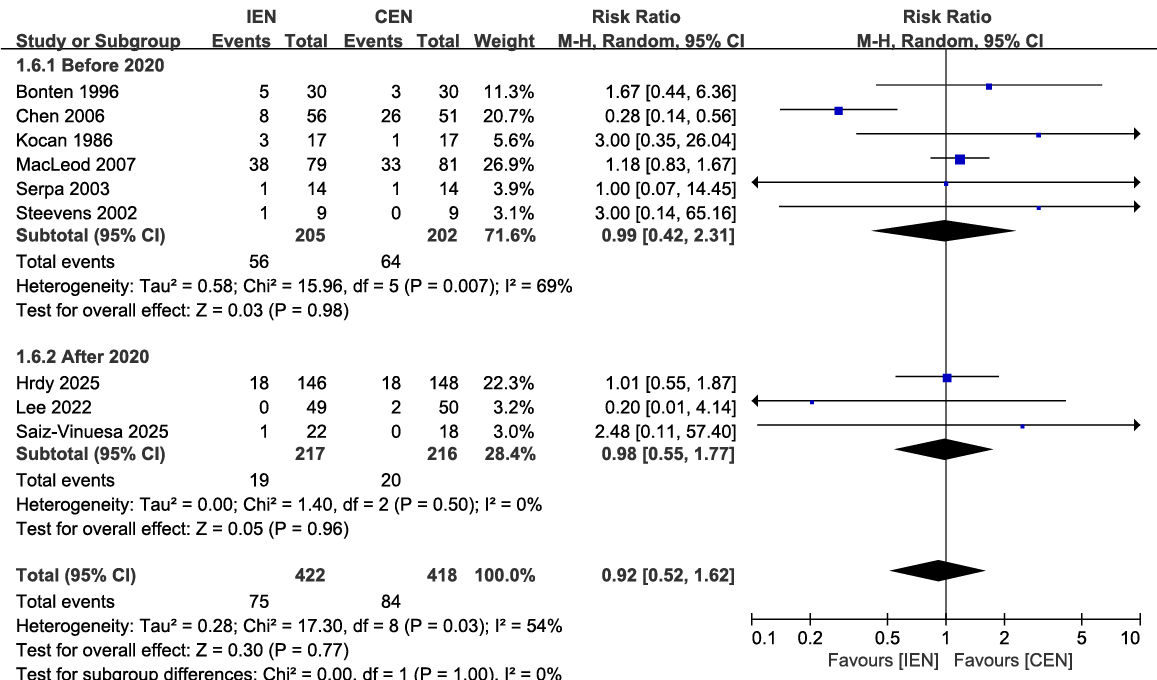


Figure 17: Subgroup analysis stratified by publication time for aspiration pneumonia


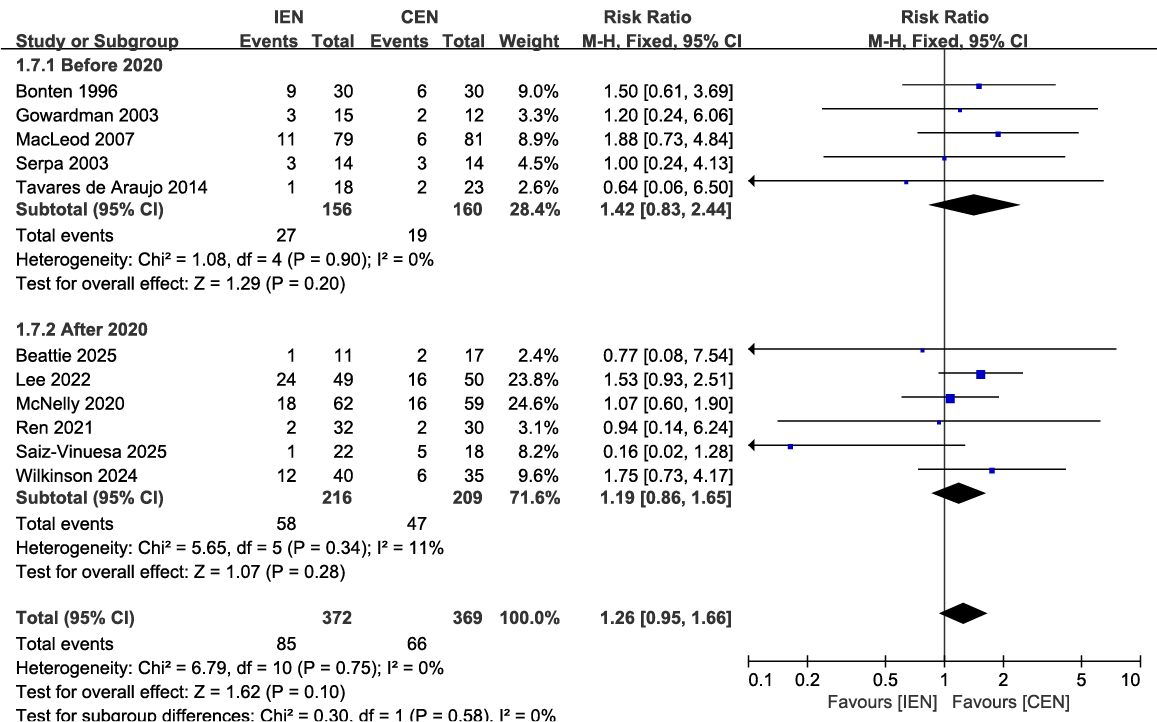


Figure 18: Subgroup analysis stratified by publication time for mortality in ICU


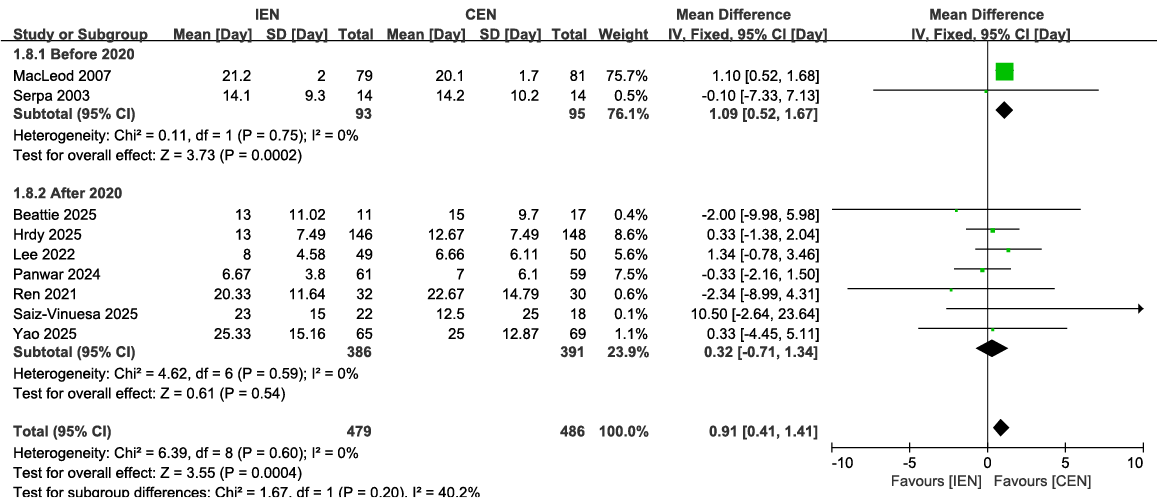


Figure 19: Subgroup analysis stratified by publication time for length of ICU stay


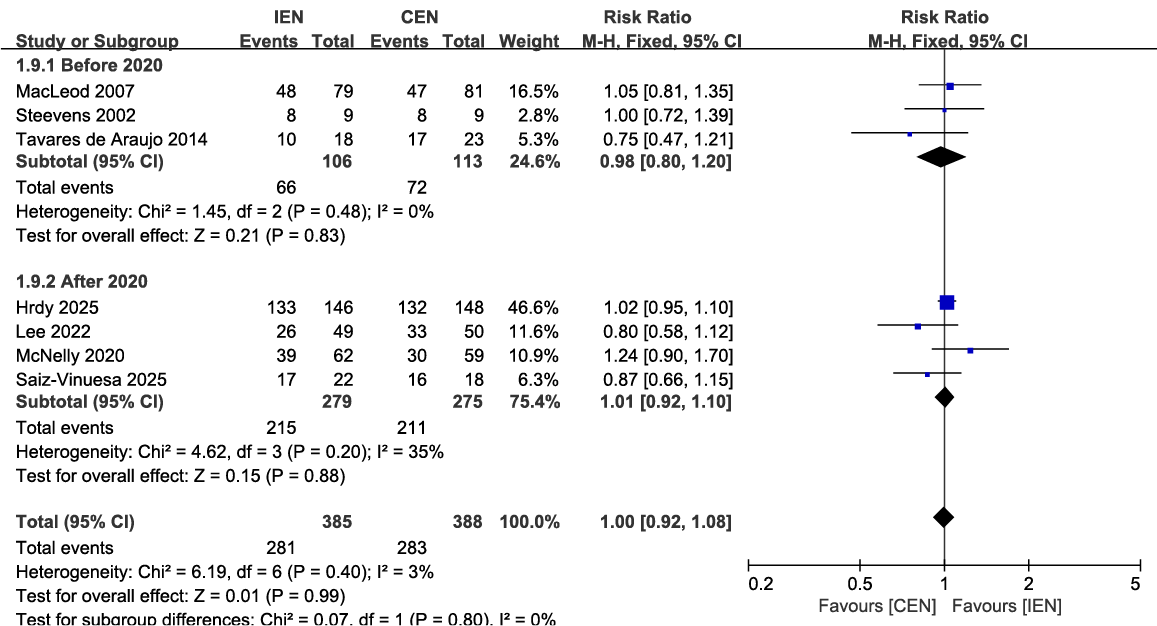


Figure 20: Subgroup analysis stratified by publication time for achievement of nutritional goal
